# Can CD147 work as a therapeutic target for tumors through COVID-19 infection?

**DOI:** 10.7150/ijms.79162

**Published:** 2022-11-21

**Authors:** Hao-Lin Ren, Gui-Min Wen, Zhen-Ying Zhao, Da-Hua Liu, Pu Xia

**Affiliations:** 1 Department of Radiology, the First Affiliated Hospital of Dalian Medical University, Dalian, Liaoning, P.R. China.; 2 Department of Basic Nursing, College of Nursing, Jinzhou Medical University, Jinzhou, Liaoning, P.R. China.; 3 Department of Pharmacy, Tianjin Union Medical Center, Tianjin, P.R. China.; 4 Biological Anthropology Institute, College of Basic Medicine, Jinzhou Medical University, Jinzhou, Liaoning, P.R. China.

**Keywords:** ferroptosis, CD147, cancer, COVID-19, SLC7A11

## Abstract

In this review, we discussed an interesting case infected with “COVID-19” (Corona Virus Disease 2019). The patients with Hodgkin's lymphoma recovered after infection with COVID-19. It may be that COVID-19 activates the patient's immune system, or it may be a coincidence. COVID-19 spike protein can interact with CD147 and use it as an entry to invade host cells. CD147 is a partner of SLC3A2, which is the chaperone subunit of cystine/glutamate reverse transporter (system XC). The catalytic subunit of system XC is SLC7A11. SLC7A11 mediated cysteine uptake plays a key role in ferroptosis. Through literature review and data analysis, we suggest that CD147, as a new potential COVID-19 infection entry, may also lead to ferroptosis of host cells. Our hypothesis is that spike protein of COVID-19 induced ferroptosis in host cells via CD147/SLC3A2/SLC7A11 complex. This is another explanation for the cancer patient recovered after COVID-19 infection.

## Introduction

Recently, a novel coronavirus pneumonia case reported in the British Journal of Hematology was that a 61 year old man suffered from lymphatic cancer and was diagnosed with novel coronavirus pneumonia 1. After four months, the virus disappeared and the man recovered from the cancer 1. The authors gave two possible reasons, one is the reaction between pathogen specific T cells and tumor antigens, the other is the activation of natural killer cells by inflammatory cytokines 1. In short, “COVID-19” (Corona Virus Disease 2019) activated the anti-tumor immune response, not only killed the new coronavirus, but also killed cancer cells. In fact, the specific mechanism is still unknown. Whether “COVID-19 kills tumor cells” is a coincidence is still under discussion. In this review, we will discuss a widely expressed marker of cancers, CD147, which is also a key protein of COVID-19 infection 2. CD147 combines with SCL3A2 (CD98hc) to form CD147-CD98hc complex to regulate metabolism and proliferation in physiological and pathological conditions 3. SLC3A2 is a single transmembrane protein, which has an intracellular N-terminal and highly glycosylated extracellular domain as the C-terminal 4. SLC7A11 is a 12 times transmembrane protein, and its N-terminal and C-terminal are located in the cytoplasm 5. These two subunits are linked by covalent disulfide bonds to form cystine/glutamate reverse transporter (system XC) 6. SLC7A11 is responsible for the main transport activity and is highly specific for cystine and glutamate 6. SLC3A2 mainly acts as chaperone to maintain the stability of SLC7A11 protein and regulate the transport of SLC7A11 to the plasma membrane 6. System XC is a sodium independent reverse transporter of cystine and glutamate 6. We not only summarized the effect of CD147 on COVID-19, but also hope to explain the mechanism of tumor inhibition of COVID-19 through CD147 and its partners.

## CD147 gene and protein

**Figure 1:**
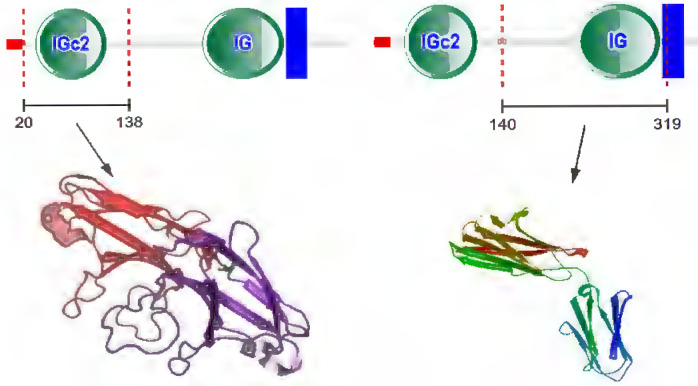
Schematic presentation of CD147 protein structure.

CD147 was first discovered in lung cancer cells by Biswas in 1982 7. It can induce fibroblasts to express collagenase, so it was named as tumor cell derived collagenase stimulating factor (TCSF) 7. It also can induce the production of multiple matrix metalloproteinases (MMPs), which was renamed EMMPRIN 8-11. CD147 has been given different names in different genera and tissues, such as EMMPRIN, TCSF, and basigin 9-11. CD147 gene is located on chromosome 19p13.3, containing 1797 bp 12. CD147 contains binding sites of specific protein 1 (SP1), activator protein 1 (AP1), transcription factor II (TFII) and early growth response factor 2 at its 5' end 12. There are two binding sites of hypoxia inducible factor (HIF) in the 3' flanking sequence 13. CD147 protein has 269 amino acid residues, which the relative molecular weight is 30-40kDa 14. The protein structure of CD147 includes N-terminal signal sequence (21 amino acid residues), extracellular immunoglobulin like domain (185 amino acid residues), single transmembrane domain (24 amino acid residues) and C-terminal intracellular domain (39 amino acid residues) 14 (Figure 1). Each domain of CD147 interacts with different proteins and plays different functions 14. The transmembrane region contains conserved hydrophobic amino acids, which can be used as the signal peptide of CD147 and the anchor site of cell membrane 14. The intracellular domain of CD147 is highly conserved and plays a key role in the interaction with the arginine residues of monocarboxylate transporter (MCT)-1 and MCT-4 15. Moreover, the transmembrane region contains a typical leucine zipper domain, which is involved in membrane protein interaction and various intracellular signaling pathways 14, 15.

## The role and the mechanism(s) of CD147 in tumor growth, metastasis and angiogenesis

**Figure 2:**
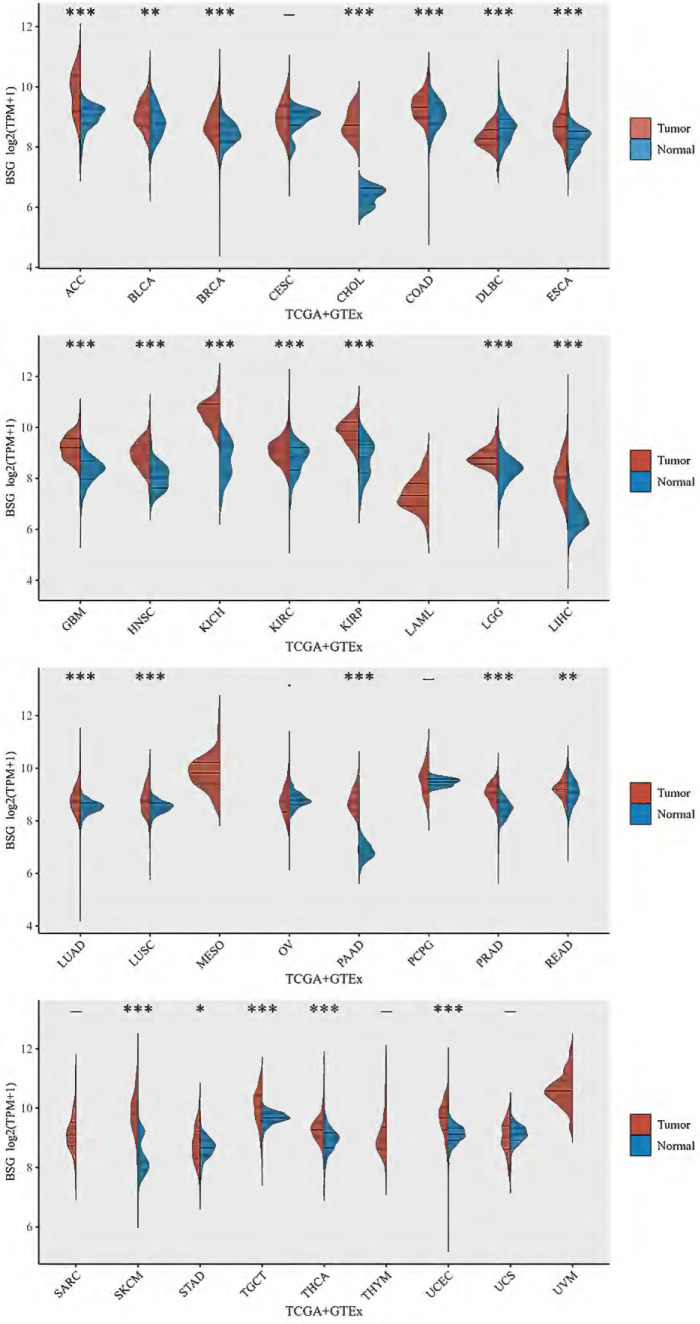
** CD147 mRNA levels in different cancer types by TCGA and GTEx database.** Abbreviations: ACC, Adrenocortical carcinoma; BLCA, Bladder Urothelial Carcinoma; BRCA, Breast invasive carcinoma; CESC, Cervical squamous cell carcinoma and endocervical adenocarcinoma; CHOL, Cholangiocarcinoma; COAD, Colon adenocarcinoma; DLBC, Lymphoid Neoplasm Diffuse Large B-cell Lymphoma; ESCA, Esophageal carcinoma; GBM, Glioblastoma multiforme; HNSC, Head and Neck squamous cell carcinoma; KICH, Kidney Chromophobe; KIRC, Kidney renal clear cell carcinoma; KIRP, Kidney renal papillary cell carcinoma; LAML, Acute Myeloid Leukemia; LGG, Brain Lower Grade Glioma; LIHC, Liver hepatocellular carcinoma; LUAD, Lung adenocarcinoma; LUSC, Lung squamous cell carcinoma; MESO, Mesothelioma; OV, Ovarian serous cystadenocarcinoma; PAAD, Pancreatic adenocarcinoma; PCPG, Pheochromocytoma and Paraganglioma; PRAD, Prostate adenocarcinoma; READ, Rectum adenocarcinoma; SARC, Sarcoma; SKCM, Skin Cutaneous Melanoma; STAD, Stomach adenocarcinoma; TGCT, Testicular Germ Cell Tumors; THCA, Thyroid carcinoma; THYM, Thymoma; UCEC, Uterine Corpus Endometrial Carcinoma; UCS, Uterine Carcinosarcoma; UVM, Uveal Melanoma.

**Figure 3:**
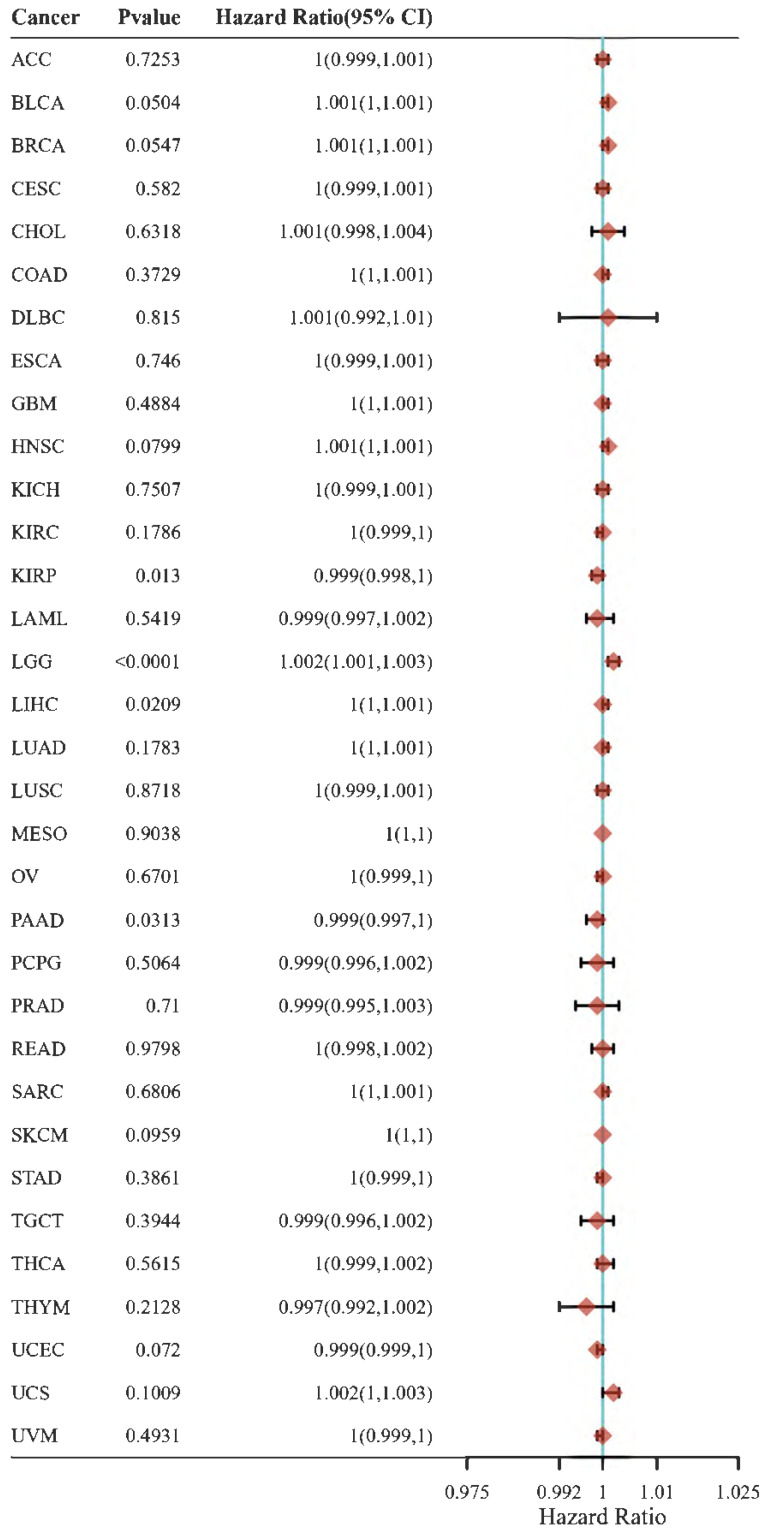
Hazard ratio of CD147 in cancers that calculated by R programming. Please refer to Figure 2 for the full name of the tumor.

CD147 is upregulated in most tumor types (Figure 2) and a hazard factor for Bladder Urothelial Carcinoma (BLCA), Brain Lower Grade Glioma (LGG), and Liver hepatocellular carcinoma (LIHC) (Figure 3). The main function of CD147 is to induce adjacent fibroblasts and endothelial cells to produce MMPs 10. CD147 rich vesicles released by tumor cells can promote the synthesis of MMPs through tumor matrix interaction 11. The secreted MMPs can degrade extracellular matrix (ECM) and promote tumor growth, invasion and metastasis 16. MMPs are secreted by CD147 stimulation, and then CD147 is removed from the cell membrane, thus forming a positive feedback loop 17. The interaction between CD147 and integrin can affect the cytoskeleton rearrangement by activating focal adhesion kinase (FAK) signaling pathway 18. The interaction between CD147 and Annexin II can inhibit the movement of hepatocellular cancer (HCC) cells, and promote the activation of SRC-dependent Rac1 signal through STAT3 19. CD147 mediates MMP dependent and independent angiogenesis 20. CD147 is up-regulated in activated human umbilical vein endothelial cells and regulates angiogenesis through secretion of MMPs 20. CD147 can induce the production of vascular endothelial growth factor (VEGF) by activating PI3K/Akt and MAPK signaling pathway 21. CD147 also can directly bind to vascular endothelial growth factor receptor 2 (VEGFR-2) and regulate VEGF mediated VEGFR-2 activation 22.

## CD147 is a potential entrance for COVID-19

**Figure 4:**
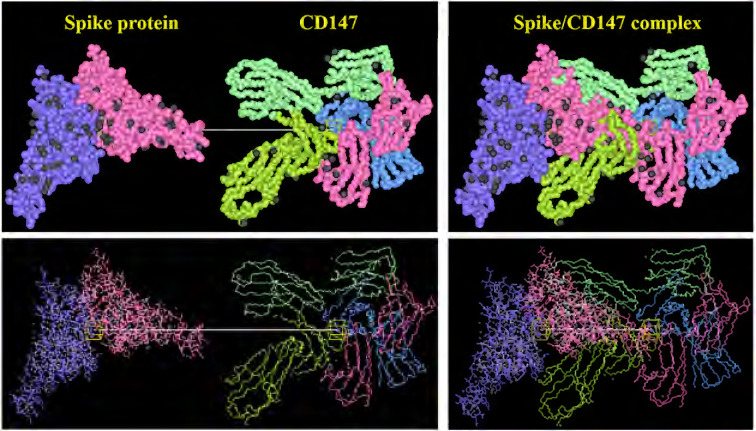
Interaction between CD147 of host cells and spike protein of COVID-19 by HEX 8.0 software.

Novel coronavirus pneumonia is a new acute respiratory infectious disease 23. Its new pathogen coronavirus (2019-nCoV or SARS-CoV-2) has the characteristics of fast propagation, wide coverage and strong infection 23. According to the data of Johns Hopkins University in the United States, as of September, 2022, more than 600 million people have been diagnosed globally 24. COVID-19 belongs to the beta coronavirus genus, and is an enveloped virus containing large RNA genome encapsulated by nucleocapsid protein (N) 25. Three transmembrane proteins were integrated into the viral lipid envelope: spike protein (S) and two smaller proteins, membrane protein (M) and envelope protein (E) 26. The spike protein of COVID-19 binds to ACE2 receptor on the surface of target cells and mediates subsequent viral uptake and fusion 27. In the process of infection, coronavirus extensively reshapes the internal membrane structure of cells and produces virus replication organelles in which virus replication takes place 27. As the first entry of COVID-19 infection, ACE2 is widely distributed in various tissues, such as heart, lung, kidney and testis, and plays an important role in controlling blood pressure, lung injury, preventing heart failure and kidney injury 28. Therefore, ACE2 as a therapeutic target may cause serious side effects. Some studies have shown that because ACE2 is widely distributed in testis, COVID-19 can affect male reproductive function 28. CD147 is a new potential way for COVID-19 to invade host cells besides ACE2 29. The combination of S protein and CD147 enhanced the invasion ability of virus to host cells (Figure 4). *Plasmodium yoelii* erythrocyte-binding-like protein (PyEBL) specifically interacts with CD147, which as the putative PyEBL receptor 30. A recent interesting study shows that in malaria endemic countries, COVID-19 and malaria parasites used CD147 as a common receptor to enter cells, resulting in a low incidence rate of COVID-19 31. All these evidences suggested CD147 is a potential entrance for COVID-19. However, it is still in debate and needs further experiments to prove.

## CD147 and ferroptosis

CD147 can inhibit autophagy by inhibiting PI3K signal pathway and down-regulating autophagy-related gene 6 (ATG6, Beclin 1) expression in ovarian cancer cells 32. Intracellular domain of CD147 (CD147-ICD) enhanced autophagy, increased mitochondria-light chain 3 (LC3) protein level, and accumulated of autophagic vesicles in hepatocellular carcinoma cells though NF κB-TRAIL-caspase8-ATG3 pathway 33. In prostate cancer cells, CD147 significantly inhibits starvation-induced autophagy via PI3K/Akt/mTOR pathway 34. Ferroptosis is a new type of programmed cell death which is iron dependent and different from apoptosis, necrosis and autophagy 35. The main mechanism of ferroptosis is that under the action of divalent iron or lipoxygenase, it catalyzes the lipid peroxidation of unsaturated fatty acids which are highly expressed on cell membrane to induce cell death; in addition, it also shows the decrease of glutathione peroxidase (GPX4) 36. The failure of GPX4 results in the accumulation of reactive oxygen species (ROS) on membrane lipids, which requires the participation of iron ions 36.

**Figure 5:**
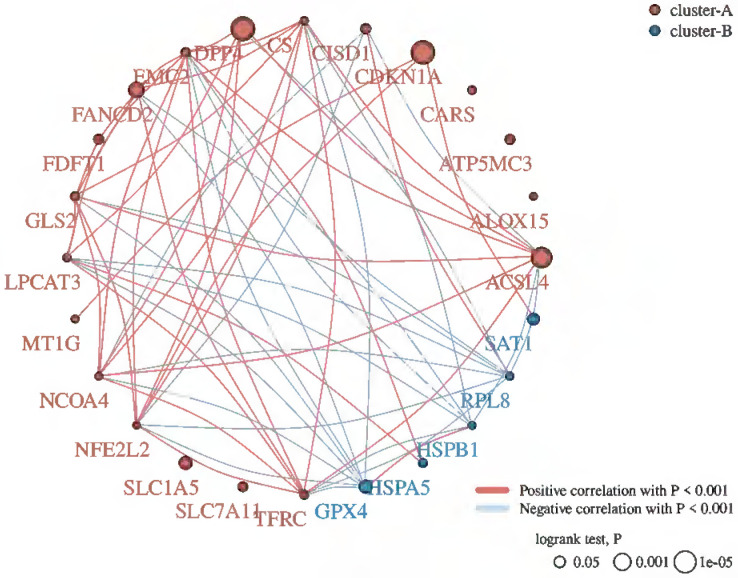
Ferroptosis related genes in Hodgkin's lymphoma by R programming.

**Figure 6:**
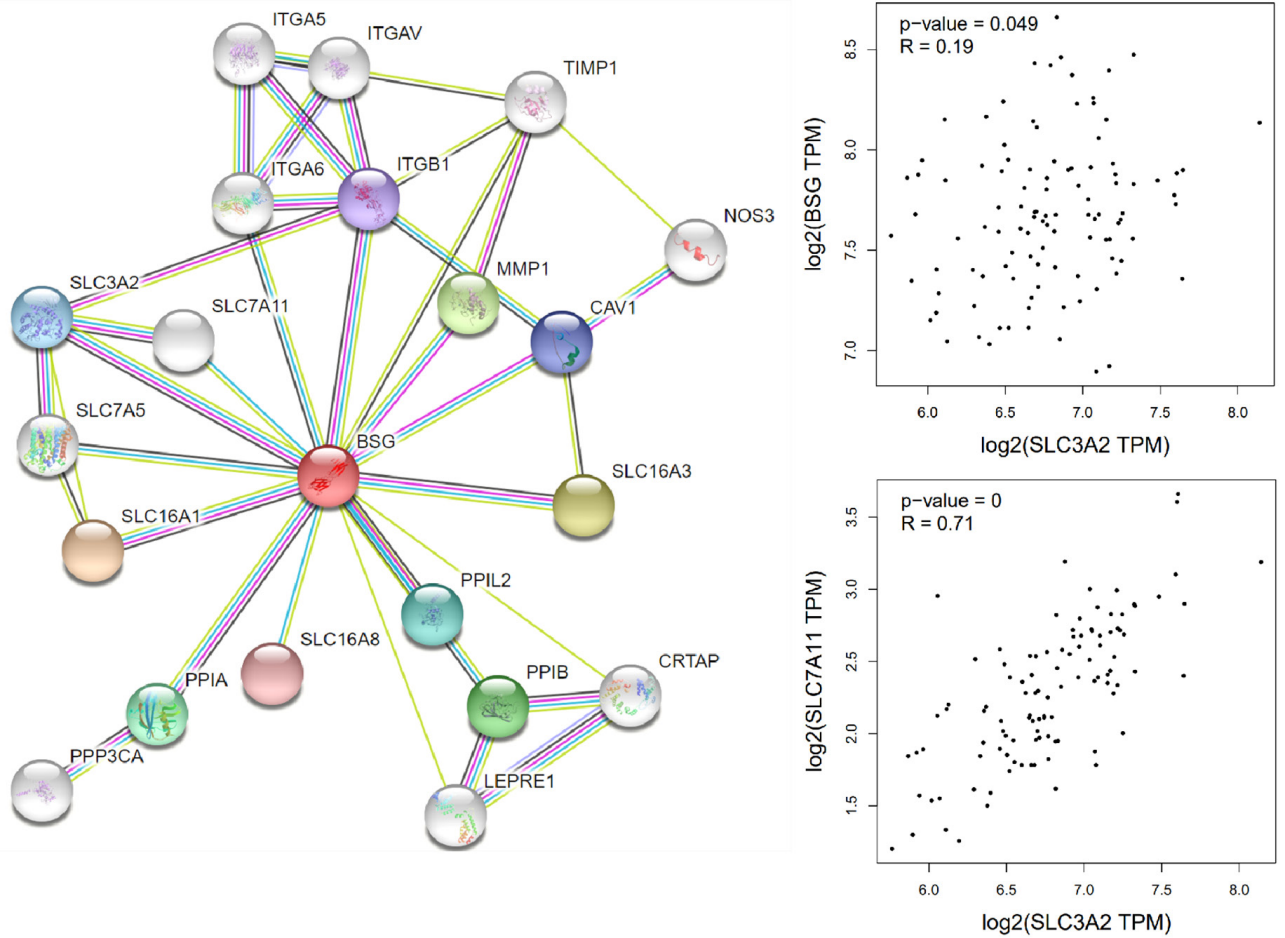
Correlation of CD147, SLC3A2 and SLC7A11 in Hodgkin's lymphoma by GEPIA (http://gepia.cancer-pku.cn/index.html).

Cells mainly rely on system XC to obtain cystine from the extracellular environment, and then convert it into cysteine in the cytoplasm through the reduction reaction of consuming NADPH 37. System XC can absorb cystine and excrete glutamate, which not only provides raw materials for intracellular glutathione synthesis, but also participates in the regulation of extracellular glutamate concentration 38. Inhibition of SLC7A11 using pharmacological inhibitors will induce ferroptosis, such as erastin or sulfasalazine 39-41. SLC7A11 gene silencing by siRNA interference also improves sensitive to erastin induced ferroptosis of cells 42. SLC7A11 mediated cysteine uptake plays a key role in inhibiting oxidative response and maintaining cell survival under oxidative stress 42. Based on the bioinformatic analysis, SLC7A11 is also a regulator for ferroptosis in EBV positive hodgkin lymphoma (HL) (Figure 5). To our knowledge, no previous study showed the interconnection of CD147 and SLC7A11. However, clear evidence demonstrated that the interaction of CD147 and SLC3A2, which is a subunit of system XC 43. In this review, we analyzed the correlation of CD147, SLC7A11 and SLC3A2 in EBV-transformed lymphocytes by the Genotype-Tissue Expression (GTEx) database (Figure 6).

## Possible mechanism(s) of COIVD-19 inhibiting tumor cells

The current hypothesis is that after COIVD-19 enters the body, the patient's immune system is activated, killing the virus, but also killing the tumor cells 1. Oncolytic virus therapy is close to this process, and currently the herpesvirus therapy has been approved for metastatic melanoma 44. In 1891, Dr. William Coley, the father of tumor immunotherapy, injected Streptococcus into two patients with osteosarcoma 45. Although the tumor also shrank, the patient died of infection 45. It is very dangerous to activate the immune system by virus. Based on our bioinformatic analysis and literature review, our hypothesis is that COIVD-19 enters into host cells through CD147, leading to partial dysfunction of CD147. CD147 can affect the transport function of SLC3A2 through SLC3A2/CD147 complex, resulting in the dysfunction of SLC7A11. However, the truth needs to be confirmed in experiments.

## Conclusion

In this review, we used ferroptosis to explain the mechanism of COIVD-19 in the treatment of tumor. As an entry of COIVD-19, CD147 can also regulate SLC7A11 through SLC3A2/CD147 complex, which is an important factor of ferroptosis. Although the corresponding mechanism can be reasonably explained in theory, it needs to be proved by experimental evidence.
